# Surface-Enhanced Raman Spectroscopy Analysis of *Astragalus* Saponins and Identification of Metabolites After Oral Administration in Rats by Ultrahigh-Performance Liquid Chromatography/Quadrupole Time-of-Flight Mass Spectrometry Analysis

**DOI:** 10.3389/fphar.2022.828449

**Published:** 2022-03-09

**Authors:** Shengnan Kong, Shan Ou, Yan Liu, Minzhen Xie, Ting Mei, Yingshuo Zhang, Jincheng Zhang, Qi Wang, Bingyou Yang

**Affiliations:** ^1^ Key Laboratory of Basic and Application Research of Beiyao (Heilongjiang University of Chinese Medicine), Ministry of Education, Heilongjiang University of Chinese Medicine, Ministry of Education, Harbin, China; ^2^ Department of Medicinal Chemistry and Natural Medicine Chemistry, College of Pharmacy, Harbin Medical University, Harbin, China

**Keywords:** astragali radix, surface-enhanced Raman spectroscopy, metabolite identification, Astragalus saponins, astragaloside IV

## Abstract

*Astragalus mongholicus* Bunge (Fabaceae) is an ancient Chinese herbal medicine, and *Astragalus* saponins are the main active components, which have a wide range of biological activities, such as immunomodulation, antioxidation, and neuroprotection. In this study, silver nanoparticles obtained by sodium borohydride reduction were used as the enhanced substrate to detect astragaloside I (1), astragaloside II (2), astragaloside III (3), astragaloside IV (4), isoastragaloside I (5), and isoastragaloside II (6) in the phloem, xylem, and cork by surface-enhanced Raman spectroscopy (SERS). In the SERS spectrum of *Astragalus* slices, the characteristic peaks were observed at 562, 671, 732, 801, 836, 950, 1,026, 1,391, and 1,584 cm^−1^, among which 950 cm^−1^ and 1,391 cm^−1^ were strong SERS signals. Subsequently, the metabolites of the six kinds of *Astragalus* saponins were identified by UPLC/ESI/Q-TOF-MS. Totally, 80, 89, and 90 metabolites were identified in rat plasma, urine, and feces, respectively. The metabolism of saponins mainly involves dehydration, deacetylation, dihydroxylation, dexylose reaction, deglycosylation, methylation, deacetylation, and glycol dehydration. Ten metabolites (1-M2, 1-M11, 2-M3, 2-M12, 3-M14, 4-M9, 5-M2, 5-M17, 6-M3, and 6-M12) were identified by comparison with reference standards. Interestingly, *Astragalus* saponins 1, 2, 5, and 6 were deacetylated to form astragaloside IV (4), which has been reported to have good pharmacological neuroprotective, liver protective, anticancer, and antidiabetic effects. Six kinds of active *Astragalus* saponins from different parts of *Astragalus mongholicus* were identified by SERS spectroscopy. Six kinds of active *Astragalus* saponins from different parts of *Astragalus mongholicus* were identified by SERS spectrum, and the metabolites were characterized by UPLC/ESI/Q-TOF-MS, which not only provided a new method for the identification of traditional Chinese medicine but also provided a theoretical basis for the study of the pharmacodynamic substance basis of *Astragalus mongholicus* saponins.

## 1 Introduction

*Astragalus mongholicus* Bunge is an ancient Chinese herbal medicine used as an essential ingredient in over 200 Chinese herbal formulas prescribed to treat different diseases in China and other Asian countries (Yuan et al., 2012; Sun et al., 2019). Pharmacological studies have shown that their saponins, flavonoids, and polysaccharide phytochemicals have interesting bioactivities, such as antioxidant, anti-inflammatory, immunomodulatory, antiviral, and antitumor activities (Liu et al., 2017). According to the Chinese Pharmacopeia (2020 version), astragaloside saponins (especially astragaloside IV) possess interesting pharmacological activities, and they are used as quality assessment markers for *Astragalus mongholicus* Bunge. At present, more than 40 triterpenoid saponins have been obtained from *Astragalus mongholicus* Bunge and its related plants (Lee et al., 2017a; Song et al., 2007).

Because *Astragalus mongholicus* Bunge has many pharmacological effects, the current research focuses on the pharmacological substance basis of *Astragalus mongholicus*, mainly including the identification and content determination of flavonoids and saponins in *Astragalus mongholicus* (Zhang et al., 2018; Mei et al., 2020). At the same time, the quality of *Astragalus mongholicus* was also determined by diffuse reflectance mid-infrared transform spectroscopy (Yang et al., 2020). In addition, several researchers have studied the pharmacokinetic characteristics of its active components and metabolites in different crude extracts (Liu et al., 2015; Shi et al., 2015). Among them, astragaloside IV has been studied for neuroprotection, liver protection, anticancer, and antidiabetes and has also been studied extensively in terms of the pharmacokinetics of rats and dogs (Cheng et al., 2016; Lee et al., 2017b; Zhang et al., 2020; Zhang et al., 2007; Zhang et al., 2005). However, *Astragalus* saponins have similar structures and more isomers, making them difficult to identify. Many potential active saponins in *Astragalus* mongholicus Bunge have not been studied.

Therefore, it is of great significance to develop a rapid and effective modern detection method for *Astragalus* saponins in chemical studies and the study of their metabolism *in vivo*. This study used SERS to detect interesting astragaloside saponins (astragaloside I (1), astragaloside II (2), astragaloside III (3), astragaloside IV (4), isoastragaloside I (5), and isoastragaloside II (6)), identify metabolites, and determine the metabolic profile of them in rat biosamples by UPLC/ESI/Q-TOF-MS to provide a fundamental basis for further pharmacology research and clinical applications of these phytochemicals.

## Materials and Methods

### Chemicals and Reagents

**FIGURE 1:**
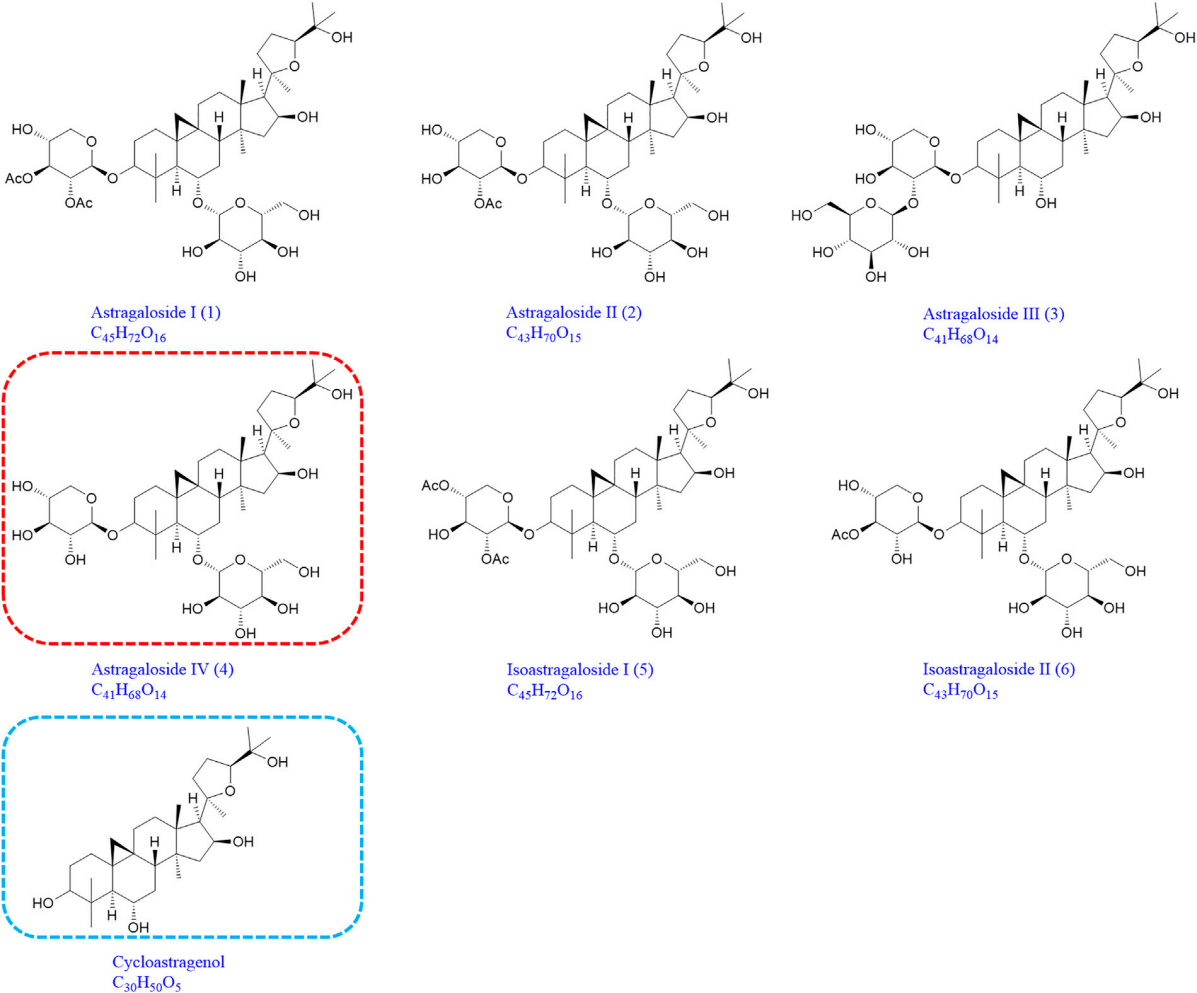
Chemical structures of astragaloside I (1), astragaloside II (2), astragaloside III (3), astragaloside IV (4), isoastragaloside I (5), isoastragaloside II (6), and cycloastragenol (the same as 1-M12, 2-M14, 3-M9, 4-M11, 5-M17, and 6-M12).

Compound astragaloside I (1), astragaloside II (2), astragaloside III (3), astragaloside IV (4), isoastragaloside I (5), isoastragaloside II (6), and cycloastragenol were all purchased from Chengdu Monster Biological Technology Co., Ltd., and their purity was above 98%. Heparin sodium was purchased from Beijing Xinyoubo Biotechnology Co., Ltd. (Figure 1). *Astragalus mongholicus* Bunge was purchased from Heilongjiang Zhongxin Co., Ltd. Sodium borohydride and silver nitrate were purchased from Aladdin. All other reagents were of analytical grade.

### Preparation of *Astragalus mongholicus* Bunge Sample and SERS Spectrum Detection

The phloem, xylem, and cork of *Astragalus mongholicus* Bunge were extracted with 0.5 g powder and 1 ml chromatographic methanol for ultrasonic extraction for 30 min. Centrifuge for 15 min at 6,000 rpm, and take the supernatant. The six *Astragalus* saponins were 1 mg, and 100 μl of chromatographic methanol was added to each and dissolved by ultrasound. 10 μl of silver nanoparticles (Ag@BO) reduced by centrifuged sodium borohydride was added into a 1.5 ml centrifuge tube; then, the sample (2 μl) was added into the centrifuge tube, mixed, and shaken well. After that, 5 μl of sodium borohydride solution was added, shaken, and mixed, and a small amount of mixed sample was absorbed by 0.5 mm capillary for Raman detection. Raman instruments are manufactured by Wetic (Germany). Raman detection parameters are as follows: laser wavelength 633 nm, grating 600, scanning time 10 s/time, laser power 10 mW, and cumulative scanning times once. All Raman signal data in this article have no other smoothing operation except for base operation.

### Animals and Drug Administration

Sixteen male Sprague Dawley rats (220–250 g) were purchased from the Experimental Animal Center of the Second Affiliated Hospital of Harbin Medical University. The laboratory animal facilities and procedures have been approved by the Animal Care and Use Committee of Harbin Medical University. All procedures are strictly implemented following the National Institute of Health Guidelines for the Care and Use of Laboratory Animals (Institute for Laboratory Animal Resources, 1996). Rats were fed in a metabolic cage with a temperature of 25°C, humidity of 60 ± 5%, 12 h dark-light cycle, free drinking water, and normal feed for three consecutive days. Rats fasted for 12 h before the experiment. Seven compounds were suspended in 1% sodium carboxymethyl cellulose to obtain suspension (2 mg/ml for each compound). Rats (*n* = 2) were orally given 40 mg/kg, while the control group was orally given the same amount of normal saline (Wang et al., 2014).

### Preparation of Plasma, Urine, and Fecal Samples

0.5 ml of ocular vein blood was collected from rats in each treatment group at 0.5, 1, 2, 4, 6, and 8 h after administration and was centrifuged at 3,000 rpm for 10 min. Plasma samples were collected from the supernatant, combined with plasma, and stored in a −20°C refrigerator for cryopreservation. Urine was collected within 24 h and stored in a refrigerator at −20°C. The feces of rats within 24 h after oral administration were collected and air-dried naturally.

1 ml of plasma was taken, and four times the volume of methanol-acetonitrile (2:1) precipitated protein was added. The precipitated protein was vortexed for 5 min and centrifuged at 13,500 rpm for 5 min, and the supernatant was taken out and rotated-dried at 37°C. 2 ml of urine was extracted and purified on an activated solid-phase extraction column (OASIS HLB 6 CC). Purification process is as follows: first eluting with 3 ml of water, then eluting with 3 ml of 5% methanol-water, and finally eluting with 5 ml of methanol. The methanol eluted parts were collected, decompressed in water at 37°C, rotated, and dried. The dry feces were ground into powder, and 1.0 g was extracted with 20 ml methanol for 30 min by ultrasonic extraction. After extraction, the supernatant was centrifugally extracted, rotated, and dried at 37°C under reduced pressure. All samples were redissolved with 200 μl methanol and filtered through a 0.45 μm filter. Triple TOF^®^ 5,600+ LC/MS/MS analysis (Xia et al., 2019).

### UPLC/ESI/Q-TOF-MS Analysis

An AB SCIEX Triple TOF 5600 was used for chromatographic separation. An ACQUITYUPLC CSHTM Phenyl-Hexyl (2.1 mm × 178,100 mm, 1.7 μm; Waters) was utilized for chromatographic separation. The mobile phase consisted of water (A) containing 0.1% (v/v) formic acid and acetonitrile (B) at a flow rate of 0.4 ml/min. The pressure limit is 15,000 psi. The linear gradient elution program was set as follows: 0–3 min, 3–30% B; 3–23 min, 30–50% B; and 23–35 min, 50–100% B. 5 μl of sample aliquot was injected onto the column, with the column temperature maintained at 35 °C. The MS full scan range was 150–1,200 m/z, and the production scan range was 80–1,000 m/z. The optimized parameters were as follows: capillary voltage, 5.5 kV; declustering potential, 80 V; and collision energy, 35 V. High-purity nitrogen (N2) and high-purity argon (Ar) were separately used as the desolvation and collision gas, respectively. The flow rate of cone gas (N2) was 0.8 L/min. The desolvation and source temperatures were 450 and 100°C, respectively. All data obtained in positive ion mode were acquired and processed by Analyst^®^ TF (V1.6) software (Xu et al., 2018).

## Results and Discussion

### Comparison in SRSE and Conventional Raman Spectra of *Astragalus mongholicus* Bunge

**FIGURE 2:**
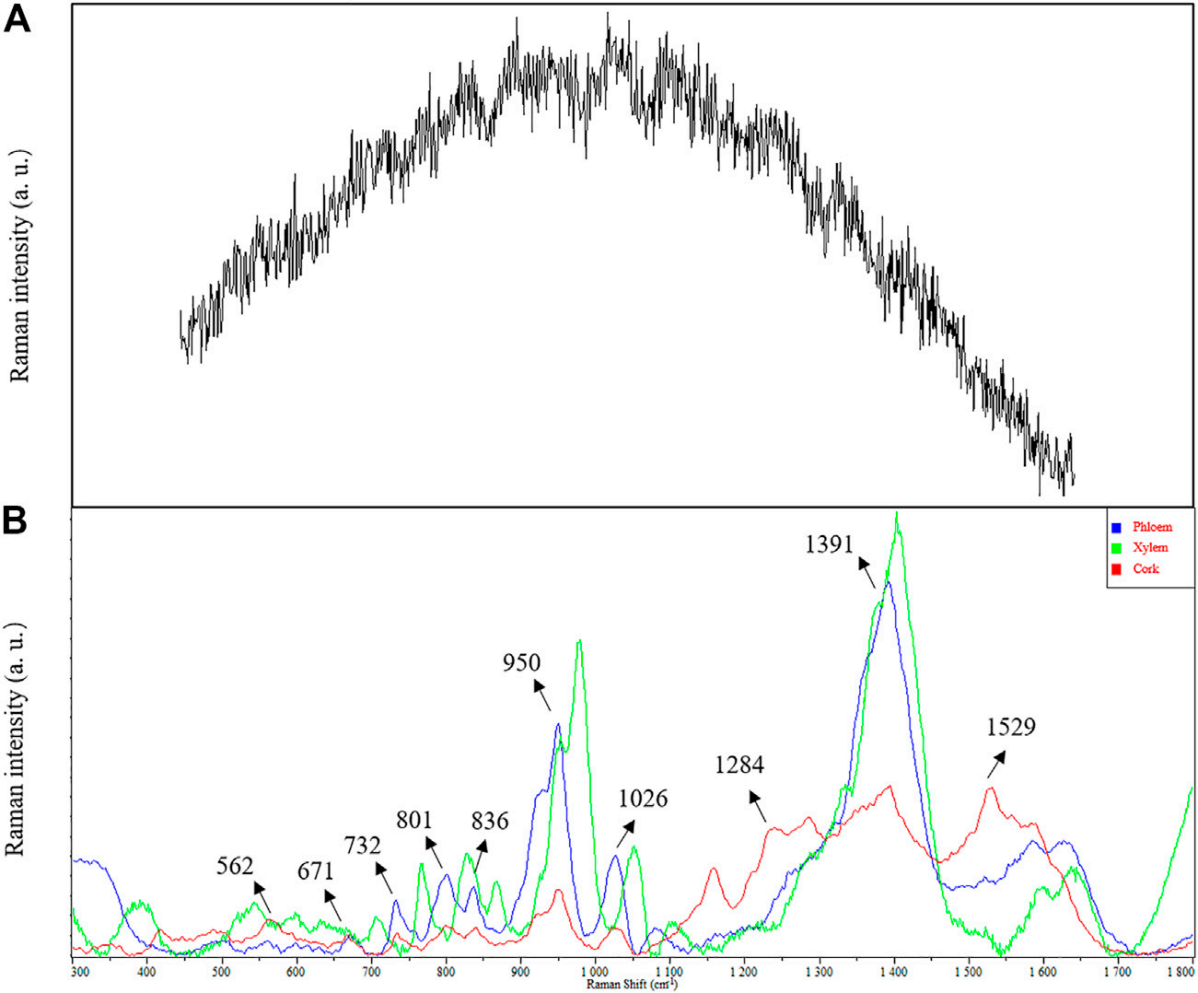
Comparison in SERS and conventional Raman spectra of *Astragalus mongholicus* Bunge. **(A)** conventional Raman spectra. **(B)** SERS.

In conventional Raman spectrum detection, the Raman effect is very weak due to strong fluorescence background interference (Figure 2A). SERS technology effectively quenched the fluorescence, and higher sensitivity was obtained by adsorbing the analyte to the plasma nanoparticles while significantly enhancing the Raman spectral signal (Figure 2B). Compared with conventional Raman spectroscopy, when noble metal nanoparticles were used, the conventional surface-enhanced Raman spectroscopy (SERS) enhanced factor was 10^6^∼10^8^ (Albrecht et al., 1977; Jeanmaire and Van Duyne, 1977; Gu et al., 2018). Therefore, SERS has become an interesting method for biological analysis of *Astragalus* and its saponins due to its excellent selectivity and high sensitivity (Stiles et al., 2008; Cialla-May et al., 2017).

### SERS Characterization of *Astragalus* Saponins

**FIGURE 3:**
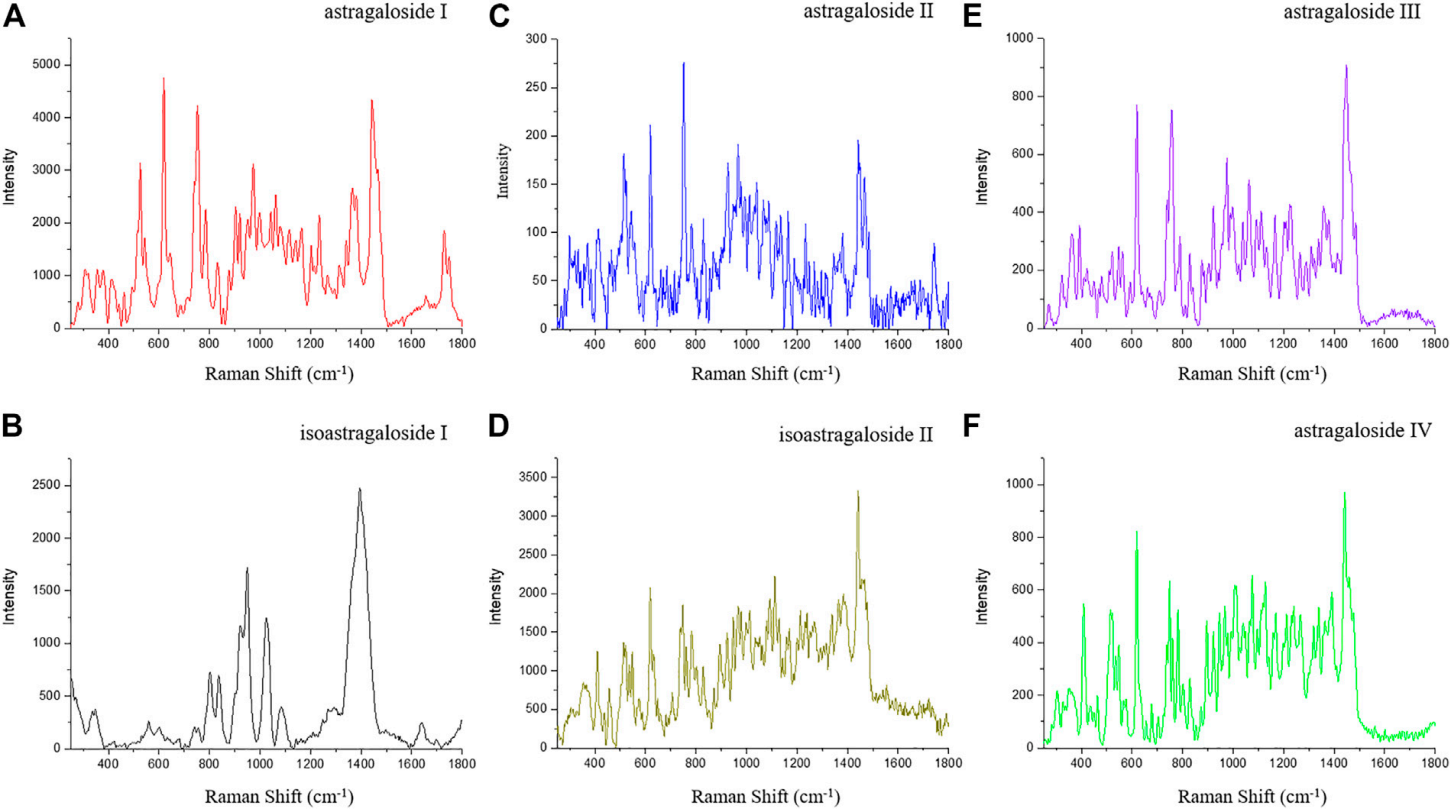
SERS of astragaloside I **(A)**, isoastragaloside I **(B)**, astragaloside II **(C)**, isoastragaloside II **(D)**, astragaloside III **(E)**, and astragaloside IV **(F)**.

*Astragalus* saponins (astragalosides I–IV and isoastragalosides I-II) have similar structures. Although they have characteristic Raman peaks at 732, 801, 836, 950, and 1,026 cm^−1^, there are significant differences (Figure 3D). Compared with the strong Raman signal peak of 1,442 cm^−1^ of other *Astragalus* saponins, the strong Raman signal of isoastragaloside I was 1,391 cm^−1^ (Figure 3B). Astragaloside I (Figures 3A) and astragaloside II (Figure 3c) were 619 cm^−1^ and 715 cm^−1^, respectively, and 1,391 cm^−1^ was the second strong signal. Astragaloside IV also had a Raman peak of 1,391 cm^−1^ (Figure 3F), but the response was very low. The strongest peak of astragaloside III shifted to the right, forming a peak of 1,448 cm^−1^ (Figure 3E).

### SERS Characterization of *Astragalus mongholicus* Bunge

Obvious Raman characteristic peaks could be observed at 562, 671, 732, 801, 836, 950, 1,026, 1,391, and 1,584 cm^−1^. 950 and 1,391 cm^−1^ were strong Raman signals, indicating the fingerprint characteristics of biochemical substances in *Astragalus mongholicus* Bunge. Due to the interaction between compounds, the Raman characteristic peaks of a compound in the prepared slices are offset from those of a single compound measured by Raman spectra. The characteristic Raman frequencies of astragaloside saponins (astragalosides I–IV and isoastragalosides I-II) were consistent with those of *Astragalus* saponins at 732, 801, 836, 950, and 1,026 cm^−1^ in *Astragalus mongholicus* Bunge Raman spectrum, indicating that *Astragalus* saponins were contained in *Astragalus* slices (Figures 2B). The peak of 1026 cm^−1^ also indicated that the contents of glycogen, amylopectin, amylose, glucuronic acid, and medium glucosamine were consistent with the known biochemical components of *Astragalus mongholicus* Bunge, such as protein, amino acid, starch, and polysaccharide. Compared with phloem and xylem, the Raman peak of 1284 cm^−1^ indicated that the cork of *Astragalus mongholicus* Bunge may contain N-acetylglucose, Deuterium N-acetylglucose, glycogen, cyclohexyl amylose, and maltose. The peak of 1529 cm^−1^ exhibits tertiary nitroalkanes, which are opposed to NO_2_ stretching. The signal intensity showed that xylem > phloem > cork.

### Characteristic Fragments of Saponins in *Astragalus mongholicus* Bunge

**FIGURE 4:**
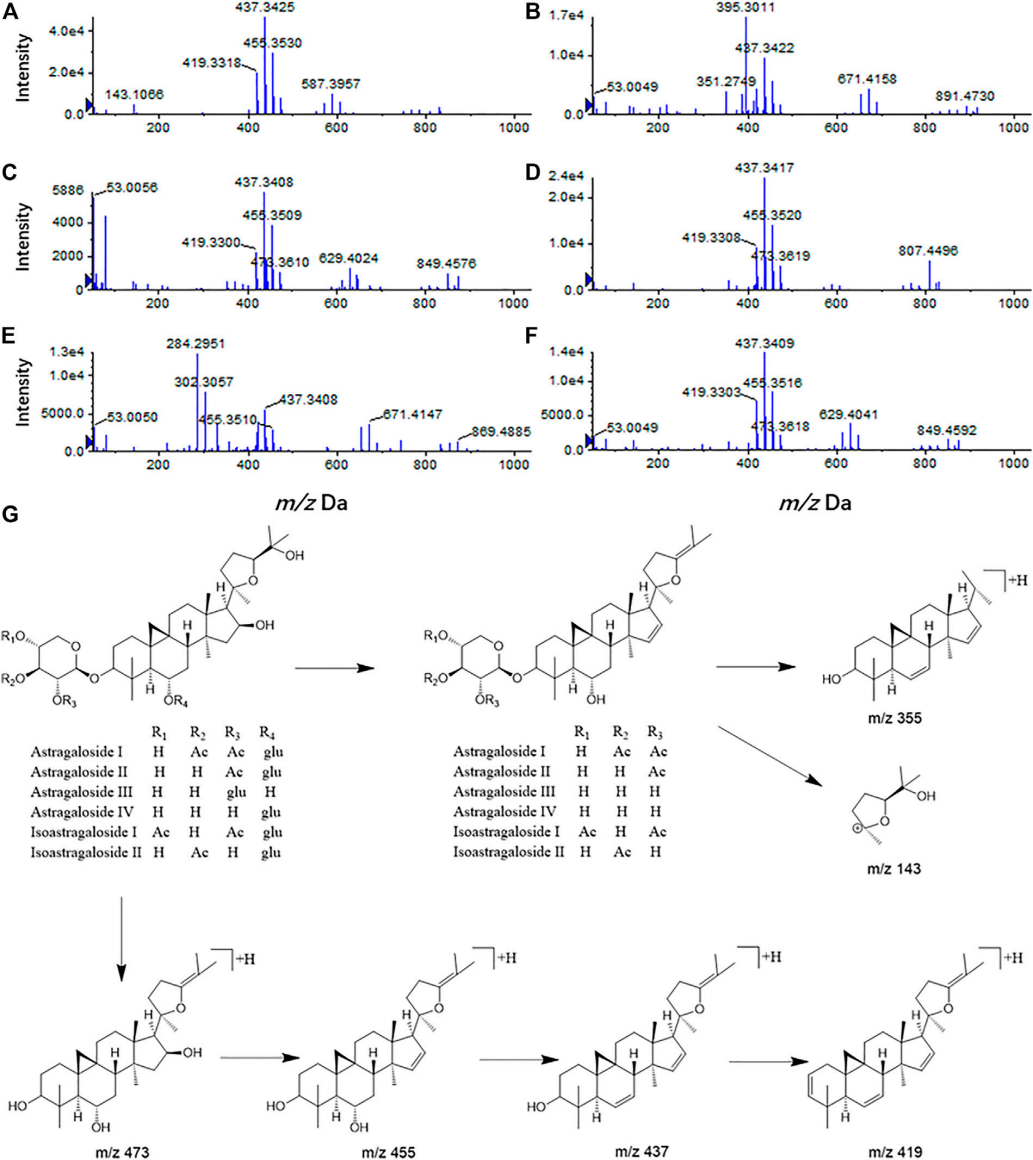
Mass spectrogram of six *Astragalus* saponins and the proposed fragmentation pathways under a positive ion mode (astragaloside I **(A)**, astragaloside II **(B)**, astragaloside III **(C)**, astragaloside IV **(D)**, isoastragaloside I **(E)**, and isoastragaloside II **(F)**, six Astragalus saponins **(G)**.

Compound 1 astragaloside I had an [M + H]^+^ peak at *m/z* 869.4880 (−2.19 ppm), and an *m/z* 671.4162 ([M + H-Glu-2*H_2_O]^+^) fragment ion was formed after the removal of glucose (162 Da) and two molecules of water (36 Da). *m/z* 473.3731, *m/z* 437.3422, and *m/z* 419.3312 were formed by continuous dehydration after aglycone formation, and *m/z* 395.3014 was formed by the loss of 84 Da (C_4_H_4_O_2_) based on *m/z* 437.3422 (Figure 4A). The [M + Na]^+^ peak of compound 2 was at *m/z* 849.4619 (0.82 ppm). In the positive ion mode, aglycones were formed with continuous water loss to produce fragments of *m/z* 455.3516, *m/z* 437.3413, and *m/z* 419.3315. In addition, after the aglycones lost two molecules of water, the five-member ring connected to C-17 lost 100 Da (C_5_H_8_O_2_) to form fragments of *m/z* 355.2631 and C-17 residue *m/z* 143.1061 ([C_8_H_15_O_2_]^+^) (Figure 4B). Compound 3 astragaloside III had an [M + H]^+^ peak at *m/z* 785.4654 (−4.20 ppm). In tandem mass spectrometry, fragments of *m/z* 587.3915, *m/z* 455.3503, *m/z* 437.3397, *m/z* 419.3296, *m/z* 355.2618, and *m/z* 143.1054 were generated (Figure 4C). High-resolution mass spectrometry showed that compound 4 astragaloside IV had an [M + Na]^+^ ion peak at *m/z* 807.4512 (0.62 ppm) in positive ion mode. In MS/MS, without glucose (162 Da) and two molecules of water (36 Da), *m/z* 587.3957 ([M + H-Glu-2 × H_2_O]^+^) was formed. In addition, under the condition of aglycone formation, four molecules of water were successively removed to form *m/z* 473.3636, *m/z* 455.3526, *m/z* 437.3422, *m/z* 419.3318, and the C-17 residue *m/z* 143.1067 ([C_8_H_15_O_2_]^+^) (Figure 4D). Compound 5 isoastragaloside I had an [M + H]^+^ peak at *m/z* 869.4871 (−3.22 ppm). The molecules generated fragments of *m/z* 671.4144, *m/z* 455.3515, *m/z* 437.3410, *m/z* 419.3303, and *m/z* 143.1059 in the secondary mass spectrometry (Figure 4E). Compound 6 isoastragaloside II had an [M + Na]^+^ peak at *m/z* 827.4785 (−0.97 ppm). Fragments of *m/z* 629.4043, *m/z* 473.3619, *m/z* 455.3515, *m/z* 437.3412, *m/z* 419.3305, and *m/z* 355.2826 were produced (Figure 4F). Compound 6 was present in large quantities in feces.

Therefore, based on the above analysis of secondary fragments of *Astragalus* saponins compounds in mass spectrometry, *Astragalus* saponins compounds can produce fragments of glucose (162 Da) and two water molecules (36 Da) in mass spectrometry. Based on lysis into aglycones, the fragments of *m/z* 473, *m/z* 455, *m/z* 437, *m/z* 419, and the aglycones were dehydrated continuously. After the loss of two molecules of water, the five-membered ring connected to C-17 lost 100 Da (C_5_H_8_O_2_) to form *m/z* 355 and the C-17 residues *m/z* 143.1061 ([C_8_H_15_O_2_]^+^) (Figure 4G).

### Metabolite Identification of *Astragalus* Saponins

**FIGURE 5:**
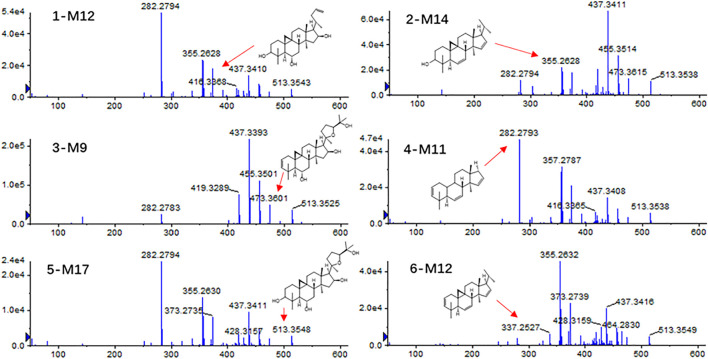
Tandem mass spectra for 1-M12, 2-M14, 3-M9, 4-M11, 5-M17, and 6-M12.

After oral administration of astragalosides I–IV and isoastragalosides I-II, compound 1 was not detected in the plasma, but it was abundant in feces. A total of 17 metabolites (1-M1 to 1-M17) were detected, among which 11 were detected in plasma and 15 in the urine and feces. In addition, compound 2 produces 18 metabolites (2-M1 to 2-M18) by desiccation, dehydration, dihydroxylation, methylation, and gluconic acid reactions, 11 of which are in the plasma and 17 of which are found in the urine and feces. Additionally, a total of 13 (3-M1 to 3-M13) metabolites with a high content were detected in the plasma, urine, and feces of rats under positive ion mode. In contrast, a total of 14 astragaloside IV-related metabolites (4-M1 to 4-M14) were detected in the rat biosamples. Among them, 4-M4 to 4-M7 were mainly detected in feces, while the remaining metabolites were identified in plasma, urine, and feces. Additionally, twenty-one metabolites (5-M1 to 5-M21) were produced in the blood, urine, and feces of rats after oral administration of isoastragaloside I. All metabolites were present in the plasma, 5-M17 was absent from the urine, and 5-M13 and 5-M14 were absent from the feces. A total of 16 (6-M1 to 6-M16) metabolites were detected in positive ion mode after oral administration of compound 6. There were 14 metabolites in the plasma and urine. Except for 6-M3, all of the other metabolites were present in feces (Figure 5).

Astragalosides II–IV and isoastragalosides I-II can cause continuous dehydration, such as metabolites 2-M1 (*m/z* 809.4675, −.48 ppm), 3-M1 (*m/z* 767.4582, −4.56 ppm), 4-M1 (*m/z* 767.4594, 1.56 ppm), 5-M4 (*m/z* 851.4793, −1.41 ppm), 6-M1 (*m/z* 809.4687, −1.73 ppm) identified as the dehydration products of *Astragalus* saponins. The six kinds of *Astragalus* saponins not only underwent deglucosylation and continuous dehydration, but also underwent dexylose reaction, deglucosylation, and continuous dehydration reactions. Furthermore, the metabolites (1-M12, 2-M14, 3-M9, 4-M11, 5-M17, and 6-M12) produced by deglycosylation of six astragalus saponins were the same (Figure 5). Except for astragaloside III, all the other saponins caused dexylose reaction, and continuous dehydration reactions occurred. Additionally, the deacetylation of astragalosides I-II and isoastragalosides I-II occurs due to the presence of acetyl groups.

The metabolite 1-M11 (*m/z* 679.405, −1.03 ppm) formed by methylation after deglycosylation of compound 1.1-M16 was a [M + Na]^+^ peak at *m/z* 501.332 (−4.99 ppm), suggesting that 1-M16 was a metabolite formed by acetylation and tri-dehydration along with the deglucosylation and dexylosylation of 1.1-M17 was determined to be C_31_H_46_O_2_ and produced *m/z* 455.3258, *m/z* 373.2740, *m/z* 355.2630, and *m/z* 318.3008 fragment ions. Hence, it was identified as a tri-dehydration and methylation product of the aglycone moiety of 1. However, 2-M16, molecular formula C_38_H_58_O_10_, is a dehydration, deglucosylation, dexylosylation methylation, and glucoaldehydation product of 2. Additionally, 3-M8 was a [M + H]^+^ peak at *m/z* 807.4493 (−4.71 ppm), suggesting that 3-M8 was a metabolite formed by glucoaldehydation and methylation along with deglycosylation and dehydration of 3. The molecular formula of 3-M13 was C_31_H_52_O_4_ (*m/z* 511.3728). It is a dehydroxylated and methylated metabolite after aglycones formed by the removal of two sugars from compound 3. In contrast, the [M + Na]^+^ peak of 5-M15 at *m/z* 849.4589 (−2.71 ppm) indicated five deacetylation, deglycosylation, methylation, and glucoaldehydation derivatives. 5-M21 was shown to be C_31_H_46_O_2_ (*m/z* 473.3364) and it is a five deacetylation, deglycosylation, dexylose reaction, dehydration, and methylation derivatives. 6-M16 was confirmed as C_32_H_48_O_4_, which is an acetylated derivative after compound 6 aglycones dehydration products (Table. 1).

**TABLE 1:** Characterization of *in vivo* metabolites of eggplant green calyx compounds 1–6 by UPLC/ESI/Q-TOF-MS.

No.	RT	Formula	Ion condition	Theoretical	Experimental	Error	MS/MS	Transformations	*p*	u	f
	(min)			(m/z)	(m/Z)	(x10^−6^)	Fragment				
1	16.29	C_45_H_72_O_16_	Na	891.4718	891.473	1.35	671.4162,473.3731,437.3422,419.3312,395.3014	Prototype		^a^	^b^
1-M1	11.33	C_43_H_70_O_15_	H	827.4793	827.4787	−0.73	629.4055,473.3630,455.3529,437.3422,419.3314,143.1064	Deacetylation	**^c^**	**^c^**	**^c^**
^d^1-M2	8.92	C_41_H_68_O_14_	Na	807.4507	807.4506	−0.12	455.3525,437.3422,355.2635,297.1854,149.0231	Double-deacetylation	**^c^**	**^c^**	
1-M3	16.42	C_43_H_70_O_14_	Na	833.4663	833.4679	1.92	437.3422,419.3313,395.3011,351.2747,201.1849,133.0856	Deacetylation/dehydroxylation		**^c^**	**^c^**
1-M4	11.30	C_36_H_60_O_10_	Na	675.4084	675.4075	−1.33	629.4054,473.2629,455.3526,437.3421,419.3313,143.1066	Double-deacetylation/dexylose reaction	**^c^**	**^c^**	**^c^**
1-M5	11.38	C_36_H_58_O_9_	H	635.4159	635.4167	1.26	629.4055,473.3632,455.3526,437.3423,419.3313,143.1066	Deacetylation/dexylose reaction/dehydration	**^c^**	**^c^**	^a^
1-M6	11.32	C_36_H_56_O_8_	H	617.4053	617.404	2.11	473.3629,455.3517,437.3419,419.3304,143.1063	Double-deacetylation/dexylcosylation/double-dehydration	**^c^**	**^c^**	**^c^**
1-M7	11.30	C_36_H_54_O_7_	H	599.3948	599.3939	-1.50	473.3629,455.3526,437.3421,419.3313,143.1066	Double-deacetylation/dexylcosylation/tri-dehydration	**^c^**	**^c^**	**^c^**
1-M8	16.37	C_39_H_60_O_10_	H	689.4265	689.4268	0.44	473.3627,437.3422,419.3310,395.3011,351.2749,217.0704	Deglucosylation/dehydration		^a^	**^c^**
1-M9	16.31	C_39_H_58_O_9_	H	671.4159	671.4151	−1.19	473.3625,455.3524,437.3420,419.3314,395.3012,217.0705	Deglucosylation/double-dehydration		^a^	^a^
1-M10	16.34	C_39_H_56_O_8_	H	653.4053	653.4057	0.61	473.3631,437.3420,419.3312,395.3013,351.2749,133.0856	Deglucosylation/tri-dehydration		^a^	**^c^**
1-M11	26.94	C_37_H_58_O_11_	H	679.4057	679.405	−1.03	635.3782,547.3257,397.3845,299.2014,149.0234	Deglucosylation/double-demethylation	**^c^**	^a^	
^d^1-M12	15.97	C_30_H_50_O_5_	Na	513.3556	513.3544	−2.34	457.3419,373.2737,355.2632,337.2524,282.2796,159.1162	Double-deacetylation/dexylcosylation/deglucosylation	^a^	^a^	**^c^**
1-M13	11.36	C_30_H_48_O_4_	H	473.3631	473.3632	0.21	455.3527,437.3423,419.3314,175.0598,143.1065	Deglucosylation/double-deacetylation/dexylcosylation/dehydration	^a^	^a^	^a^
1-M14	11.28	C_30_H_46_O_3_	H	455.3525	455.3529	0.88	437.3422,419.3313,285.0760,175.0600,143.1065	Deglucosylation/double-deacetylation/dexylcosylation/double-dehydration	^a^	^a^	^a^
1-M15	11.25	C_30_H_44_O_2_	H	437.342	437.342	0.00	419.2212,285.0758,175.0594,143.1062	Double-deacetylation/dexylcosylation/deglucosylation/tri-dehydration	^b^	^a^	^a^
1-M16	10.56	C_32_H_46_O_3_	Na	501.3345	501.332	−4.99	473.3262,391.2841,373.2737,355.2631	Double-deacetylation/dexylcosylation/deglucosylation/tri-dehydration/acetylation			^b^
1-M17	8.34	C_31_H_46_O_2_	Na	473.3396	473.3372	−5.07	455.3258,373.2740,355.2630,318.3008	Double-deacetylation/dexylcosylation/deglucosylation/tri-dehydration/methylation			^b^
2	11.35	C_43_H_70_O_15_	Na	849.4612	849.4619	0.82	473.3622,455.3516,437.3413,419.3315,355.2631,143.1061	Prototype	**^c^**	^a^	^b^
2-M1	11.33	C_43_H_68_O_14_	H	809.4687	809.4675	−1.48	635,4,176,611.3944,473.3626,455.3519,437.3410,419.3306,401.3192,297.2240,143.1057	Dehydration		**^c^**	**^c^**
2-M2	19.95	C_43_H_66_O_13_	H	791.4582	791.4588	0.76	655.2749,629.4038,537.2873,437.3411,419.3308,373.2735,355.2633,317.2475,143.1060	Double-dehydration		**^c^**	**^c^**
^d^2-M3	8.98	C_41_H_68_O_14_	H	785.4687	785.4677	−1.27	587.3940,473.3620,455.3520,437.3413,419.3306,373.2736,355.2631,337.2524,143.1061	Deacetylation		^a^	^a^
2-M4	12.92	C_41_H_68_O_13_	Na	791.4558	791.4575	2.15	473.3362,455.3514,437.3414,373.2736,355.2635,143.1060	Deacetylation/dehydroxylation		**^c^**	**^c^**
2-M5	11.16	C_36_H_60_O_10_	Na	675.4084	675.4073	−1.63	635.4174,599.3935,473.3622,455.3519,437.3413,419,3,306,401.3202,143.1059	Deacetylation/dexylcosylation	**^c^**	**^c^**	^a^
2-M6	11.25	C_36_H_58_O_9_	H	635.4159	635.4177	2.83	473.3619,455.3513437.3412,419.3303,143.1060	Deacetylation/dexylcosylation/dehydration		**^c^**	^a^
2-M7	11.21	C_36_H_56_O_8_	H	617.4053	617.4039	−2.27	473.3617,455.3518,437,3,413,373.2735,355.2630,143.1062	Deacetylation/dexylcosylation/double-dehydration	**^c^**	**^c^**	^a^
2-M8	11.33	C_36_H_54_O_7_	H	599.3948	599.3927	−3.50	473.3618,455.3520,437.3415,419.3307,143.1067	Deacetylation/dexylcosylation/tri-dehydration	**^c^**	^a^	^a^
2-M9	12.95	C_37_H_58_O_9_	H	647.4159	647.4131	−4.32	629.4038,537.2873,437.3411,419.3308,373.2735,355.2633,317.2475	Deglucosylation/dehydration		^a^	^a^
2-M10	11.38	C_37_H_56_O_8_	H	629.4053	629.404	−2.07	473.3624,455.3519,437.3409,373.2732,355.2631,337.2532,143.1042	Deglucosylation/double-dehydration	**^c^**	^a^	^a^
2-M11	12.95	C_37_H_54_O_7_	H	611.3948	611.3936	−1.96	455.3511,437.3411,373.2735,355.2633,317.2475	Deglucosylation/tri-dehydration		^a^	^a^
2-M12	11.44	C_35_H_58_O_9_	Na	645.3979	645.3957	−3.41	629.4030,473.3611,455.3511,437.3407,419.3300,297.2208,240.2323,175.0598	Deacetylation/deglucose	**^c^**	**^c^**	
2-M13	12.98	C_35_H_58_O_8_	Na	629.4029	629.4044	2.38	557.3788,455.3506,437.3411,373.2735,355.2632,317.2476,219.1738	Deacetylation/dehydroxylation/deglucosylation	**^c^**		^a^
^d^2-M14	15.97	C_30_H_50_O_5_	Na	513.3556	513.3537	−3.70	473.3611,455.3511,437.3408,419.3304,355.2628,282.2795,143.1065	Deacetylation/dexylcosylation/deglucosylation	**^c^**	**^c^**	^a^
2-M15	11.18	C_30_H_48_O_4_	H	473.3631	473.3618	−2.75	455.3519,437.3414,419.3309,401.3197,143.1059	Deglucosylation/deacetylation/dexylcosylation/dehydration	^a^	^a^	^a^
2-M16	11.13	C_38_H_58_O_10_	H	675.4108	675.4071	−5.48	473.3620,455.3520,437.3403,419.3308,401.3198,143.1058	Deglucosylation/deacetylation/dexylcosylation/dehydration/glucoaldehydation/methylation	**^c^**	**^c^**	^a^
2-M17	11.21	C_30_H_46_O_3_	H	455.3525	455.3517	−1.76	437.3412,419.3307,401.3195143.1058	Deglucosylation/deacetylation/dexylcosylation/double-dehydration	^a^	^a^	^b^
2-M18	11.23	C_30_H_44_O_2_	H	437.342	437.3413	−1.60	419.3307,389.2679,371.2577,353.2470,143.1061	Deacetylation/dexylcosylation/deglucosylation/tri-dehydration	^b^	^b^	^b^
3	9.27	C_41_H_68_O_14_	Na	807.4507	807.4496	−4.20	587.3915,455.3503,437.3397,419.3296,355.2618,143.1054	Prototype	^a^	^a^	^a^
3-M1	9.32	C_41_H_66_O_13_	H	767.4582	767.4547	−4.56	587.3912,473.3604,455.3503,437.3399,419.3292,373.2723,355.2618,2143.1054	Dehydration	**^c^**	^a^	^a^
3-M2	9.35	C_41_H_64_O_12_	H	749.4476	749.4435	−5.47	587.3918,455.3505,437.3399,419.3292,373.2721,355.2612,143.1054	Double-dehydration	**^c^**	**^c^**	^a^
3-M3	9.24	C_41_H_62_O_11_	H	731.437	731.4339	−4.24	587.3918,473.3606,455.3502,437.3397,419.3291,373.2723,355.2618,337.2515,143.1054	Tri-dehydration	**^c^**	**^c^**	**^c^**
3-M4	9.21	C_35_H_58_O_9_	H	623.4159	623.4142	−2.73	587.3939,473.3620,455.3518,437.3412,419.3305,143.1063	Deglucosylation	**^c^**	**^c^**	
3-M5	9.19	C_35_H_56_O_8_	H	605.4053	605.4016	−6.11	473.3606,455.3502,437.3395,419.3291,371.2565,143.1056	Deglucosylation/dehydration	**^c^**	^a^	^a^
3-M6	9.15	C_35_H_54_O_7_	H	587.3948	587.3936	−2.04	473.3620,455.3518,437.3411,419.3306,245.0474	Deglucosylation/double-dehydration	**^c^**	^a^	
3-M7	9.23	C_35_H_52_O_6_	H	569.3842	569.3829	−2.28	473.3621,455.3517,437.3413,419.3307,143.1060	Deglucosylation/tri-dehydration	**^c^**	**^c^**	
3-M8	9.17	C_43_H_66_O_14_	H	807.4531	807.4493	−4.71	473.3626,455.3521,437.3416,419.3307,355.2631,143.1065	Deglucosylation/dehydration/glucoaldehydation/methylation	^a^	**^c^**	^a^
^d^3-M9	15.98	C_30_H_50_O5	Na	513.3556	513.3551	−3.05	473.3620,455.3518,437.3412,419.3308,143.1061	Dexylcosylation/deglucosylation	**^c^**	**^c^**	^a^
3-M10	11.30	C_30_H_48_O_4_	H	473.3631	473.3607	−5.07	455.3504,437.3398,419.3293,373.2724,355.2618,143.1054	Deglucosylation/dexylcosylation/dehydration	^a^	^a^	^b^
3-M11	11.19	C_30_H_46_O_3_	H	455.3525	455.3503	−4.83	437.3399,419.3293,282.2781,373.2723,355.2617,143.1054	Deglucosylation/dexylcosylation/double-dehydration	^a^	^b^	^b^
3-M12	11.25	C_33_H_44_O_2_	H	437.342	437.3401	−4.34	389.2671,371.2569,355.2622,331.2263	Dexylcosylation/deglucosylation/tri-dehydration	^b^	^b^	^b^
3-M13	8.73	C_31_H_52_O_4_	Na	511.3763	511.3728	−6.84	448.3055,430.2952,412.2847,363.3271,355.2635,219.1741	Dexylcosylation/deglucosylation/dehydroxylation/methylation	^a^		**^c^**
4	9.08	C_41_H_68_O_14_	Na	807.4507	807.4512	0.62	587.3957,473.3636,455.3526,437.3422,419.3318,143.1067	Prototype	^a^	^a^	^a^
4-M1	9.24	C_41_H_66_O_13_	H	767.4582	767.4594	1.56	587.3956,569.3856,473.3636,455.3530,437.3424,419.3319,373.2750,355.2635	Dehydration	**^c^**	**^c^**	^a^
4-M2	8.90	C_41_H_64_O_12_	H	749.4476	749.448	0.53	587.3953,569.3842,473.3633,455.3529,437.3423,419.3320,373.2743,355.2638	Double-dehydration	**^c^**	**^c^**	^a^
4-M3	8.98	C_41_H_62_O_11_	H	731.437	731.437	0.00	473.3637,455.3529,437.3425,419.3319,401.3207,297.2217,143.1068	Tri-dehydration	**^c^**	**^c^**	**^c^**
4-M4	11.17	C_36_H_60_O_10_	Na	675.4081	675.4061	0.30	473.3614,455.3514,437.3408,419.3302,371.2575,143.1063	Dexylcosylation			^a^
4-M5	11.23	C_36_H_58_O_9_	H	635.4159	635.4151	1.26	455.3527,437.3423,419.3314,355.2640,143.1062	Dexylcosylation/dehydration			^a^
4-M6	11.14	C_36_H_56_O_8_	H	617.4053	617.4035	2.92	473.3634,455.3530,437.3425,419.3320,143.1065	Dexylcosylation/double-dehydration			^a^
4-M7	11.20	C_36_H_54_O_7_	H	599.3948	599.3935	2.17	473.3636,455.3531,437.3425,419.3317,355.2641,143.1067	Dexylcosylation/tri-dehydration			^a^
4-M8	9.17	C_35_H_56_O_8_	H	605.4053	605.4035	−2.97	587.3937,569.3828,473.3618,455.3515,437.3409,419.3303,373.2735,355.2627	Deglucosylation/dehydration	^a^	^a^	^a^
4-M9	8.96	C_35_H_54_O_7_	H	587.3948	587.3933	−2.55	455.3515,437.3410,419.3302,389.2681,373.2735,355.2628,335.2365	Deglucosylation/double-dehydration	^a^	^a^	^b^
4-M10	9.00	C_35_H_52_O_6_	H	569.3842	569.385	1.41	473.3632,455.3531,437.3426,419.3317,143.1068	Deglucosylation/tri-dehydration	^a^	^a^	^a^
^d^4-M11	15.96	C_30_H_50_O_5_	Na	513.3556	513.3538	−3.51	455.3510,437.3408,416.3365,357.2787,282.2793	Dexylcosylation/deglucose	^a^	^a^	^a^
4-M12	9.19	C_30_H_48_O_4_	H	473.3631	473.3635	0.85	455.3531,437.3424,419.3316,389.2685,355.2645,143.1067	Deglucosylation/dexylcosylation/dehydration	^a^	^a^	^a^
4-M13	9.36	C_30_H_46_O_3_	H	455.3525	455.3516	−1.98	437.3410,389.2683,373.2735,355.2630,335.2365,271.1691	Deglucosylation/dexylcosylation/double-dehydration	^b^	^b^	^b^
4-M14	9.06	C_30_H_44_O_2_	H	437.342	437.3425	1.14	419.3320,355.2640,341.1055,245.0479,143.1068	Dexylcosylation/deglucosylation/tri-dehydration	^b^	^b^	^b^
5	17.77	C_45_H_72_O_16_	H	869.4899	869.4871	−3.22	671.4144,455.3515,437.3410,419.3303,143.1059	Prototype	^a^	^a^	^a^
5-M1	14.29	C_43_H_70_O_15_	Na	849.4612	849.46	−1.41	647.4132,611.3921,473.3593,437.3404,357.2786,318.3001,161.1317	Deacetylation	**^c^**	**^c^**	**^c^**
^d^5-M2	9.09	C_41_H_68_O_14_	Na	807.4507	807.4496	−1.36	767.4565,617.4022,587.3934,473.3614455.3514,437.3408,419.3301,355.2625,297.2207,143.1058	Double-deacetylation	**^c^**	**^c^**	^a^
5-M3	17.86	C_43_H_70_O_14_	Na	833.4663	833.4667	0.48	741.4411,689.4251,671.4145,455.3514,437.3411	Deacetylation/dehydroxylation	**^c^**	^a^	**^c^**
5-M4	17.72	C_45_H_70_O_15_	H	851.4793	851.4781	−1.41	741.4411,689.4251,671.4145,455.3514,437.3411,330.3005,302.3056,284.2951	Dehydration	**^c^**	^a^	**^c^**
5-M5	17.88	C_45_H_66_O_13_	H	815.4582	815.4584	0.25	741.4411,689.4251,671.4145,455.3514,437.3411,330.3005,302.3056,284.2951	Tri-dehydration	**^c^**	**^c^**	**^c^**
5-M6	11.13	C_36_H_60_O_10_	Na	675.4084	675.4067	−2.52	473.3616,455.3517,437.3411,419.3302,143.1061	Double-deacetylation/dexylcosylation	**^c^**	**^c^**	^a^
5-M7	11.31	C_36_H_58_O_9_	H	635.4159	635.4151	−1.26	473.3615,455.3515,437.3410,419.3302,355.2622,143.1061	Double-deacetylation/dexylcosylation/dehydration	**^c^**	^a^	^a^
5-M8	11.34	C_36_H_56_O_8_	H	617.4053	617.4044	−1.46	473.3619,455.3516,437.3410.419.3304,401.3195,143.1062	Double-deacetylation/dexylcosylation/double-dehydration	**^c^**	**^c^**	^a^
5-M9	11.18	C_36_H_54_O_7_	H	599.3948	599.3924	−4.00	473.3617,455.3515,437.3409,419.3304,143.1062	Double-deacetylation/dexylcosylation/tri-dehydration	**^c^**	**^c^**	^a^
5-M10	17.75	C_39_H_60_O_10_	H	689.4265	689.4247	−2.61	671.4145,437.3410,355.2628,330.3001,302.3054,284.2947,217.0698	Deglucosylation/dehydration	**^c^**	**^c^**	**^c^**
5-M11	17.80	C_39_H_58_O_9_	H	671.4159	671.4145	−2.09	653.4039,455.3514,437.3411,330.3005,302.3056,284.2951,284.2951,217.0697	Deglucosylation/double-dehydration	^a^	^a^	^a^
5-M12	17.83	C_39_H_56_O_8_	H	653.4053	653.404	−1.99	455.3510,437.3408,419.3303,355.2627,330.3005,302.3057,284.2951,217.0698	Deglucosylation/tri-dehydration	^a^	^a^	^a^
5-M13	14.97	C_37_H_60_O_10_	Na	687.4084	687.4063	−3.05	566.3233,460.2692,415.2111,318.3001,267.1221	Deacetylation/deglucosylation	**^c^**	**^c^**	
5-M14	15.02	C_35_H_58_O_9_	Na	645.3979	645.3979	0.00	503.1080,429.0882,318.3008,219.1739	double-deacetylation/deglucosylation	**^c^**	**^c^**	
5-M15	11.42	C_43_H_70_O_15_	Na	849.4612	849.4589	−2.71	698.4831,639.4035,473.3615,455.2519,437.3411,419.3304,373.2726,143.1060	Double-deacetylation/deglucosylation/glucoaldehydation/double-methylation	**^c^**	**^c^**	**^c^**
5-M16	17.81	C_37_H_60_O_9_	Na	671.4135	671.4149	2.09	635.3962,455.3518,437.3411,419.3307,217.0701	Deacetylation/dehydroxylation/deglucosylation	^a^	^a^	^a^
^d^5-M17	15.97	C_30_H_50_O_5_	Na	513.3556	513.3539	−3.31	455.3517,437.3411,419.3303,373.3736,355.2628,337.2528,143.1059	Double-deacetylation/dexylcosylation/deglucosylation	**^c^**		^a^
5-M18	11.26	C_30_H_48_O_4_	H	473.3631	473.3613	−3.80	455.3518,437.3412,419.3305,401.3198,353.2467,175.0593,143.1061	Deglucosylation/double-deacetylation/dexylcosylation/dehydration	**^c^**	^a^	^a^
5-M19	11.20	C_30_H_46_O_3_	H	455.3525	455.3516	−1.98	437.3410,419.3303,401.3196,371.2577,355.2570,143.1059	Deglucosylation/double-deacetylation/dexylcosylation/double-dehydration	^a^	^a^	^b^
5-M20	11.15	C_30_H_44_O_2_	H	437.342	437.341	−2.29	419.3302,401.3196,297.3306,143.1061	Double-deacetylation/dexylcosylation/deglucosylation/tri-dehydration	^a^	^a^	^b^
5-M21	8.40	C_31_H_46_O_2_	Na	473.3396	473.3364	−6.76	413.3042,371.2574,355.2629,318.3006	Double-deacetylation/dexylcosylation/deglucosylation/tri-dehydration/methylation	^a^	**^c^**	**^c^**
6	11.34	C_43_H_70_O_15_	Na	849.4612	849.4592	−0.97	629.4043,473.3619,455.3515,437.3412,419.3305,355.2826	Prototype	**^c^**	**^c^**	^a^
6-M1	12.90	C_43_H_68_O_14_	H	809.4687	809.4673	−1.73	629.4045,473.3621,455.3518,437.3412,419.3309,401.3197,297.2210,143.1062	Dehydration	**^c^**	**^c^**	^a^
6-M2	11.58	C_43_H_66_O_13_	H	791.4582	791.457	−1.52	473.3621,455.3520,437,3414,419,3,307,371.2581,317.2477,143.1063	Double-dehydration		**^c^**	^a^
^d^6-M3	8.93	C_41_H_68_O_14_	Na	807.4507	807.4487	−2.48	587.3937,473.3622,455.3515,437.3412,419.3306,355.2628,143.1058	Deacetylation	**^c^**	**^c^**	
6-M4	11.42	C_41_H_68_O_13_	Na	791.4558	791.4567	1.14	647.4149,629.4047,611.3941,473.3622,455.3521,437.3416,419.3308,355.2631,175.0594,143.1062	Deacetylation/dehydroxylation	**^c^**	**^c^**	^a^
6-M5	11.15	C_36_H_60_O_10_	Na	675.4084	675.4071	−1.92	635.4183,480.3137,455.3516,437.3408,419.3306	Deacetylation/dexylcosylation	**^c^**	**^c^**	^a^
6-M6	11.17	C_36_H_58_O_9_	H	635.4159	635.4179	3.15	455.3514,437.3407,419.3305,335.2379,143.1061	Deacetylation/dexylcosylation/dehydration	**^c^**	**^c^**	^a^
6-M7	11.12	C_36_H_56_O_8_	H	617.4053	617.4046	−1.13	534.3410,498.2881,473.3619,455.3519,437.3409,419.3307,389.2684,335.2572,143.1061	Deacetylation/dexylcosylation/double-dehydration	**^c^**	**^c^**	^a^
6-M8	11.31	C_37_H_58_O_9_	H	647.4159	647.4153	−0.93	629.4047,611.3940,473.3618,455.3512,437.3414,419.3308,373.2731,355.2630,175.0596,143.1060	Deglucosylation/dehydration	^a^	^a^	^a^
6-M9	11.39	C_37_H_56_O_8_	H	629.4053	629.4047	−0.95	473.3622,455.3520,437.3414,419.3308,355.2628,175.0591,143.1060	Deglucosylation/double-dehydration	^a^	^a^	^a^
6-M10	11.28	C_37_H_54_O_7_	H	611.3948	611.3939	−1.47	473.3621,455.3519,437.3413,419.3307,371.2579,355.2631,175.0594,143.1062	Deglucosylation/tri-dehydration	^a^	^a^	^a^
6-M11	11.36	C_35_H_58_O_8_	Na	629.4029	629.4044	2.38	473.3620,455.3519,437.3413,419.3308,355.2624,175.0591,143.1059	Deacetylation/dehydroxylation/deglucosylation	^a^	^a^	^a^
^d^6-M12	15.99	C_30_H_50_O_5_	Na	513.3556	513.3549	−1.36	455.3518,437.3416,373.2739,355.2632,337.2527	Deacetylation/dexylcosylation/deglucosylation	**^c^**		^a^
6-M13	11.25	C_30_H_48_O_4_	H	473.3631	473.3622	−1.90	455.3519,437.3413,419.3307,401.3197,297.2210,143.1061	Deglucosylation/deacetylation/dexylcosylation/dehydration	^a^	^a^	^b^
6-M14	11.23	C_30_H_46_O_3_	H	455.3525	455.3518	−1.54	437.3414,419.3309,401.3194,297.2208,143.1062	Deglucosylation/deacetylation/dexylcosylation/double-dehydration	^a^	^b^	^b^
6-M15	11.21	C_30_H_44_O_2_	H	437.342	437.3414	−1.37	419.3307,401.3202,297.2213,143.1056	Deacetylation/dexylcosylation/deglucosylation/tri-dehydration	^b^	^b^	^b^
6-M16	6.70	C_32_H_48_O_4_	Na	519.345	519.3431	−3.66	389.2686,371.2579,333.2425	Deglucosylation/deacetylation/dexylcosylation/double-dehydration/Acetylation			^b^

d Identified by comparing with reference standards.

b Detected at the highest abundance.

a detected at comparatively high abundance.

c detected at detected at low abundance (+++>++>+).

*p* plasma; u: urine; f: feces.

### Metabolic Pathways of Compounds 1–6 in *Astragalus mongholicus* Bunge

*Astragalus* saponins 1–6 could not easily be used as a prototype component in plasma after oral administration, but they had a higher content in feces (Fu et al., 2019). This may be related to the physical and chemical properties of saponins, such as their high molecular weight, high hydrogen bondability, polymer flexibility, and poor membrane permeability, leading to a decrease in their bioavailability (Gao et al., 2012; Yu et al., 2012). Among the phase I metabolites, a relatively large number of dehydration products can be observed in biological samples, all of which are formed by the dehydration of hydroxyl groups at the 3, 6, and 16 positions, which is consistent with our previous findings (Cheng and Wei, 2014; Chen et al., 2018; Lü et al., 2019). Compounds 1, 2, 5, and 6 also had diacetyl metabolites in their biological samples due to the presence of one or two acetyl substitutions on glucose. In addition, compounds 1, 2, and 5 had glycolaldehyde and methylation and acetylation of compound 6, which are all unique metabolic forms but have not been reported previously (Wan et al., 2016; Chen et al., 2019; Li et al., 2021).

**FIGURE 6:**
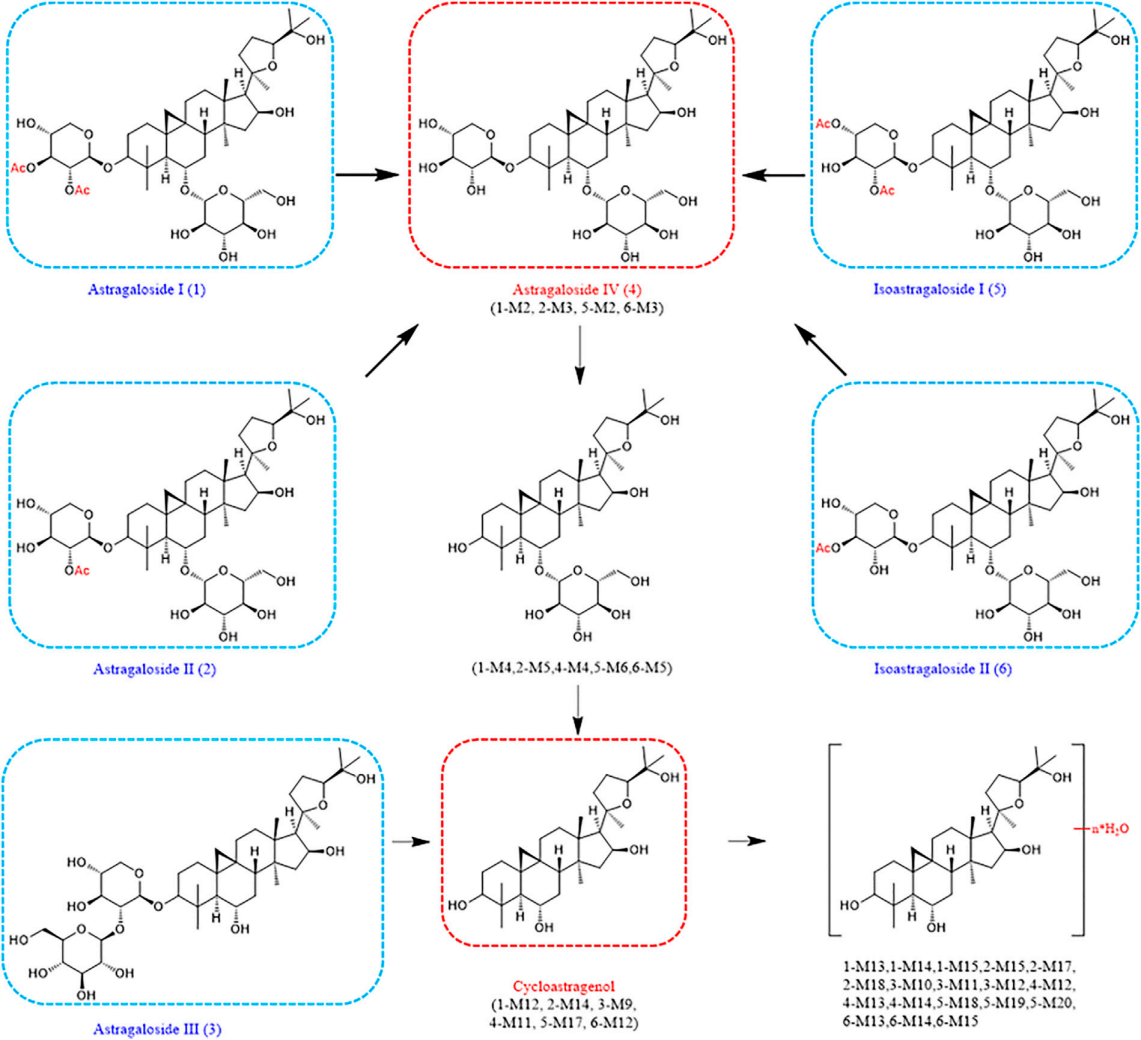
Metabolic pathways of compounds 1–6 in *Astragalus mongholicus* Bunge.

In identifying metabolites of astragaloside IV, 12 new dehydration products were added compared with the previously known ones (Cheng and Wei, 2014). Other pharmacological activities of *Astragalus* saponins have been less studied, all of which were the first metabolites studied here. Interestingly, these six *Astragalus* saponins all underwent deglycosylation and dexylose reaction reactions in the body to form the same aglycones that then underwent dehydration. More importantly, astragalosides I-II and isoastragalosides I-II can form astragaloside IV, which has increased activity after removing the acetyl group (Figure 6). This leads us to speculate that astragalosides I-II and isoastragalosides I-II may have the same potential anti-inflammatory, antifibrotic, antioxidative stress, antiasthma, antidiabetes, immunoregulation, and cardioprotective effects as astragaloside IV (Li et al., 2017).

## Conclusion

In this study, silver nanoparticles obtained by sodium borohydride reduction were first used as the enhanced substrate to detect astragaloside I (1), astragaloside II (2), astragaloside III (3), astragaloside IV, (4) isoastragaloside I (5), and isoastragaloside II (6) in the phloem, xylem, and cork, by SERS. The Raman signal and mass spectrometry decomposition of the detection results were analyzed. In the SERS spectrum of astragalus slices, the characteristic peaks were observed at 562, 671, 732, 801, 836, 950, 1,026, 1,391, and 1,584 cm^−1^, among which 950 cm^−1^ and 1,391 cm^−1^ were strong SERS signals. The SERS peak locations obtained could be attributed to biochemical substances such as *Astragalus* saponin, glucose, and acetamide. The technology of SERS can be used as a new, quick, and effective detection method for biochemical analysis, quality control, and discrimination of decocting pieces of *Astragalus mongholicus* Bunge or other Chinese medicine. UPLC/ESI/QTOF-MS was used to detect six representative *Astragalus* saponins in biological samples after the oral administration of *Astragalus mongholicus* Bunge to rats. Their metabolites were identified, and their metabolic pathways and transformation formed *in vivo* were summarized. The metabolism of *Astragalus* saponins 1–6 mainly involved dehydration, deacetylation, dihydroxylation, deglycosylation, methylation, deacetylation, and glycol dehydration reactions. Ten metabolites were identified by comparison with reference standards. According to earlier studies, this is the first study on the metabolism of these *Astragalus* saponins. The most valuable is that astragalosides I-II and isoastragalosides I-II can form astragaloside IV with better activity after removing acetyl groups. This study is of great significance for applying SERS spectroscopy in the identification of TCM and predicting the metabolism of other saponins with similar structures in *Astragalus mongholicus* Bunge, which can promote the systematic study of multi-component metabolism and clinical efficacy of the *Astragalus mongholicus* Bunge.

## Data Availability

The original contributions presented in the study are included in the article/Supplementary Material, Further inquiries can be directed to the corresponding authors.
